# Predicting Allosteric Effects from Orthosteric Binding in Hsp90-Ligand Interactions: Implications for Fragment-Based Drug Design

**DOI:** 10.1371/journal.pcbi.1004840

**Published:** 2016-06-02

**Authors:** Arun Chandramohan, Srinath Krishnamurthy, Andreas Larsson, Paer Nordlund, Anna Jansson, Ganesh S. Anand

**Affiliations:** 1 Department of Biological Sciences, National University of Singapore, Singapore; 2 School of Biological Sciences, Nanyang Technological University, Singapore; University of North Texas System College of Pharmacy, UNITED STATES

## Abstract

A key question in mapping dynamics of protein-ligand interactions is to distinguish changes at binding sites from those associated with long range conformational changes upon binding at distal sites. This assumes a greater challenge when considering the interactions of low affinity ligands (dissociation constants, K_D,_ in the μM range or lower). Amide hydrogen deuterium Exchange mass spectrometry (HDXMS) is a robust method that can provide both structural insights and dynamics information on both high affinity and transient protein-ligand interactions. In this study, an application of HDXMS for probing the dynamics of low affinity ligands to proteins is described using the N-terminal ATPase domain of Hsp90. Comparison of Hsp90 dynamics between high affinity natural inhibitors (K_D_ ~ nM) and fragment compounds reveal that HDXMS is highly sensitive in mapping the interactions of both high and low affinity ligands. HDXMS reports on changes that reflect both orthosteric effects and allosteric changes accompanying binding. Orthosteric sites can be identified by overlaying HDXMS onto structural information of protein-ligand complexes. Regions distal to orthosteric sites indicate long range conformational changes with implications for allostery. HDXMS, thus finds powerful utility as a high throughput method for compound library screening to identify binding sites and describe allostery with important implications for fragment-based ligand discovery (FBLD).

## Author Summary

Ligand interactions with proteins result in broad changes that are propagated throughout the target proteins, across space and time. These changes can be broadly classified into: orthosteric effects at the ligand binding site and allosteric changes at distal sites. These allosteric changes are difficult to localize and distinguish from binding interactions. In this study, we describe the application of amide hydrogen/deuterium exchange mass-spectrometry (HDXMS) to differentiate between changes occurring at the binding site and at distal allosteric sites by combining HDXMS with X-ray crystallography. Every ligand or a fragment mediates distinct contacts and results in changes in deuterium uptake across the protein. By comparing with orthosteric structural information, it is possible to identify long-range changes (action at a distance) due to the ligands. An important application of HDXMS is that it can identify subtle changes in protein dynamics that cannot be picked up by quantitative screens of protein-ligand interactions or crystal structures. This gives us the ability to describe ligand binding based on the response from different regions in the proteins. Thus it provides us with the potential to accurately measure and compare changes in dynamics upon binding different ligands and fragments, which is greatly valuable in fragment-based ligand design.

## Introduction

Ligands mediate specific interactions with proteins and alter their conformational dynamics thereby modulating their function, making them important regulators of biological processes [1]. Protein-ligand interaction strengths range from weak (Dissociation constants, K_D_ μM~mM) to strong affinities (K_D_ ~nM-pM) and are governed both by association and dissociation kinetics [2]. Screening for high affinity ligands, mapping their interactions and identifying the mode of ligand binding to proteins is consequently critical for small molecule inhibitor design. X-ray crystallography has been a powerful method of choice for obtaining high resolution structures of ligand-protein complexes and provide atomic level insights of ligand interactions and their binding sites. However, these only represent snapshot average endstates of proteins that do not always provide a complete overview of long range conformational changes. These represent observed changes that are distal from the proximal ligand binding sites as defined by high resolution structures. A comprehensive picture of the effects of ligand interactions comes from dynamics measurements of protein-ligand interactions in solution. Here, dynamics is defined as the average measured output of inherent fluctuations, motions and conformational rearrangements of the protein. A comparison of dynamics of protein in the absence and presence of ligand allows for a detailed description of interactions of the ligand both at the site of binding (orthosteric) and accompanying long range conformational changes with allosteric implications [3]. A major question that remains is how proximal binding effects at orthosteric sites can be distinguished from long-range conformational changes at distal sites across the protein.

Allostery is defined as communication between non-contiguous distal sites on proteins through structural and energetic changes [4]. Allostery is closely associated with an ensemble view of protein conformations wherein allosteric phenomena can be described through processes with variable dynamics ranging from local unfolding and intrinsic disorder to rigid body motions [5]. Dynamics is consequently fundamental to an understanding of allostery [6, 7]. In this study, we report how mass spectrometry offers a powerful tool for protein dynamics that can complement well-established structural biological techniques such as X-ray crystallography and Nuclear Magnetic Resonance (NMR) spectroscopy by enabling screening and mapping of low affinity ligand interactions. Mass spectrometry offers numerous tools to complement structural biology such as amide hydrogen/deuterium exchange mass spectrometry (HDXMS), crosslinking, ion mobility, hydroxyl footprinting, limited proteolysis and native mass spectrometry [8, 9].

**Fig 1:**
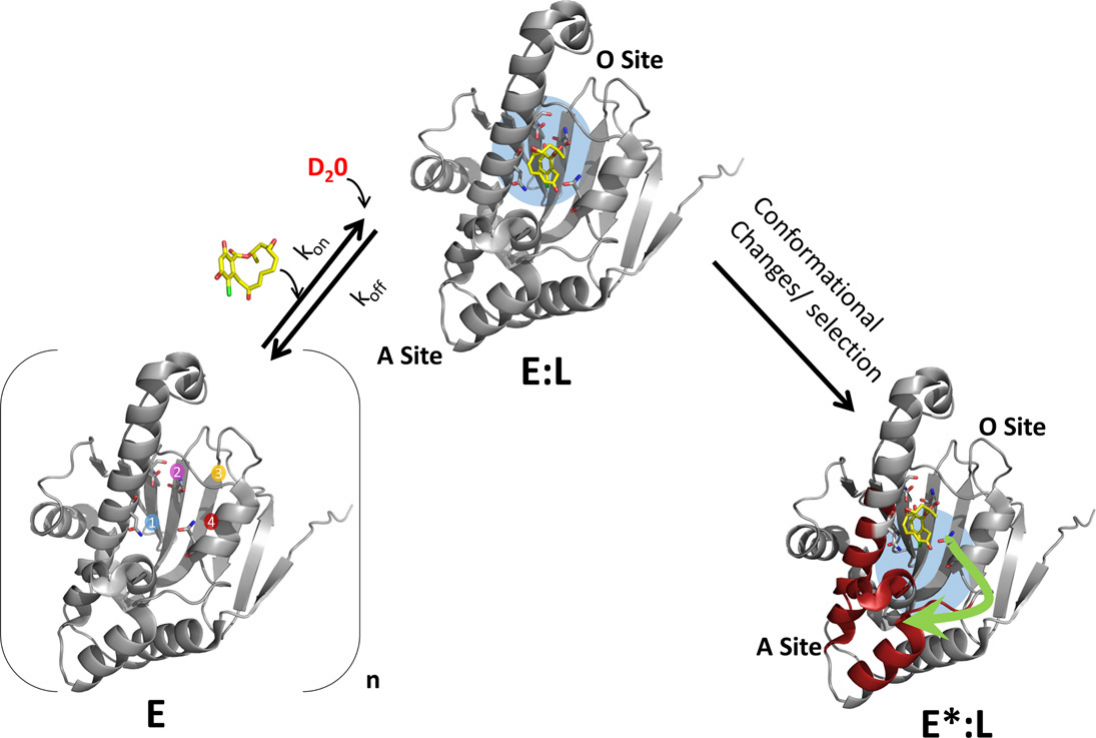
Mapping protein-ligand interactions by HDXMS. Protein-ligand interactions can be analyzed by HDXMS by comparing deuterium exchange of the unliganded state of the protein with that bound to ligand (shown in yellow sticks). An ensemble view entails that the target protein **(E)** would exist in multiple conformations in the absence of ligand. Here a representative target protein is shown containing two sites- an orthosteric **(O)** site forming the ligand binding pocket (sites 1–4 are represented) and an allosteric **(A)** site. Deuterium exchange at the orthosteric site (O-site) (blue) is then governed by ligand binding kinetic parameters: kon, koff, concentration of ligand as well as the observed rate of HDX exchange, kex, which varies across different regions of the protein. The HDXMS output encompasses changes at the orthosteric O-site and long range conformational changes (red) at the allosteric A-site. Binding of ligand at the O-site **(E:L)** would result in decreased exchange while changes at the A-site (E*:L) could be reflected as decreases or increases in deuterium exchange.

HDXMS in particular, has proven to be a powerful technique for monitoring protein dynamics in solution explainable by changes in the rates of hydrogen bonding (H-bond breakage and structural unfolding) [10] or through changes in solvent accessibility (solvent penetration model)[11] or a combination of both effects[12]. One of the important applications of HDXMS is as a comparative tool to monitor effects of diverse perturbations on proteins at peptide resolution and associated conformational changes. Changes in HDXMS thus provide a readout of the dynamics of proteins encompassing the gamut of inherent fluctuations, local unfolding, rigid body motions and conformational rearrangements. This makes it a sensitive tool for perturbation analysis. The most common perturbations examined include ligand and protein-protein interactions. Ligands mediate reversible non-covalent interactions. Changes in deuterium exchange upon ligand or protein interactions would therefore be dependent on both dynamics and kinetics of the protein-ligand interactions. Upon ligand binding, HDXMS would report both orthosteric and long range conformational changes associated with allosteric sites (Fig 1). Combining the results of HDXMS with X-ray crystallography of protein-ligand complexes, can enable identification of those HDXMS changes associated with ligand binding, from the ligand- residue contacts at the active site from the crystallographic structure. We define long range conformational changes at distal sites as those that are observed in peptides upon ligand binding, where none of the residue atoms lie within H-bonding radius (> 4Å) of any ligand atom, and also not identified as binding sites in the structure (PDB entry). Regions showing HDXMS changes that do not correlate with those regions associated with orthosteric binding are denoted as long range allosteric effects.

In protein-protein interactions, decreases in deuterium exchange at interfaces have been attributed primarily to protection from solvent access [13–16]. In the case of protein-ligand interactions, ligands being much smaller, shifts in deuterium exchange upon binding are attributed primarily to shielding of H-bonds by ligand [17]. In the context of protein-ligand interactions therefore, hydrogen bonding would play a greater role than solvent accessibility at orthosteric binding sites. A basis for changes in deuterium exchange due to shielding of solvent from interfaces in protein-protein interactions has been described in great detail and the effects observed are dependent on an interplay of the rates of association, dissociation, ligand concentrations together with the intrinsic rate of exchange [14]. This is equally relevant for protein-ligand interactions at orthosteric sites. Interactions of proteins with weak affinity ligands represents an important challenge and consequently only a limited application of HDXMS to characterize interactions between proteins and low affinity fragment molecules has been attempted [18], (Anand and Krishnamurthy patent filing). Fragment based ligand design (FBLD) has, in recent years, proven to be an attractive alternative approach to traditional high-throughput screening (HTS) techniques to develop inhibitors against therapeutically important target proteins. Fragments are small (< 300 Da), largely hydrophilic molecules that are derived from the breakdown of large ligand molecules [19, 20]. Fragments constitute the active moieties of large ligand molecules and serve as the building blocks to form larger and tighter binding molecules. Due to their small sizes they typically mediate weak protein-fragment interactions with target proteins and hence are difficult to characterize. A rule of three, to define fragments has been proposed which additionally limits fragments as molecules containing ≤ 3 H-bond donors and acceptors [21]. Fragments have previously been shown to induce conformational changes upon binding to target proteins [22] thus implying that they are capable of mediating highly specific interactions with target proteins [23]. A major challenge in FBLD consequently is detecting low affinity interactions, especially at the initial stages for identification of lead fragment molecule identification [19].

In this study, we have tested the suitability of HDXMS for identifying time-dependent changes elicited by binding of fragments and high affinity ligands to proteins using the N-terminal ATPase domain of the chaperone Hsp90 (Heat Shock Protein 90). This domain has been extensively characterized as a target for drug discovery and FBLD [24–28]. High affinity inhibitors of Hsp90 have been identified and isolated from natural sources, namely Geldanamycin and Radicicol, which bind to the N-terminal ATPase domain. In this study we report the comparative dynamics analysis of the interactions of two high affinity ligands, Radicicol (K_D_~19 nM) and a Geldanamycin derivative 17-N-Allylamino-17-demethoxygeldanamycin (17-AAG) (K_D_ ~ 33 nM) and two low affinity fragments phenolic compounds, Methyl 3,5-Dihydroxyphenylacetate (Fragment **1**) and 2,4 Dihydroxypropiophenone (Fragment **2**) [26] with binding affinities of ~500 μM with the N-terminal ATPase domain of Hsp90.

We further describe a workflow design to monitor low affinity protein-fragment interactions with an opportunity to priority rank compounds for preferential development and also distinguish orthosteric and allosteric sites with both strong and weak affinity inhibitors by overlaying HDXMS results with high resolution crystallographic structures of protein-ligand complexes. Our results show that HDXMS can indeed detect and quantitate interactions of low affinity inhibitors with proteins, which has important implications for FBLD. Further, HDXMS in combination with X-ray crystallography can allow categorization of different series of compounds based on the type of responses they elicit into orthosteric and allosteric binders. This has profound implications for FBLD.

## Materials and Methods

### Experimental optimization and design: HDXMS at protein-ligand binding sites

A primary consideration before carrying out an HDXMS experiment for mapping protein-ligand interactions is to determine optimal concentrations of protein and ligand necessary for the HDXMS experiment. A theoretical overview for mapping protein-protein interfaces has been provided previously in great detail [14]. The primary consideration is to ensure high enough concentration of the partner protein or ligand is used under deuterium exchange conditions to ensure that all the target protein is fully bound in the complexed state. To further summarize that study, experimental measurement of deuterium exchange at amides from solvent-excluded interface regions by the observed rate of deuterium exchange (k_obs_) is an interplay of dissociation rates (k_off_), association rates (k_on_) and concentration of the binding protein (Pr-ligand) as described in Eq 1 where k_ex_ is the intrinsic rate of exchange. [14]

$${\text{k}}_{\text{obs}}=\frac{{\text{k}}_{\text{off}}\times {\text{k}}_{\text{ex}}}{{\text{k}}_{\text{on}}[\text{Pr}-\text{ligand}]+{\text{k}}_{\text{ex}}}$$ (1)

Three scenarios are described in the fully bound complexes: 1) For protein-protein complexes, where the dissociation rates are very slow, k_obs_ is entirely dependent upon k_off_, k_off_ is very slow and indicates that the complex would not dissociate during the time course of a deuterium exchange experiment. 2) For protein-protein complexes with dissociation rates faster than 10^−2^ min^-1^ and with dissociation constants > 10 nM, the complex would dissociate during the HDX experiment timescales (minutes to hours) and an estimate of the observed rate of exchange would be only possible with a knowledge of all of the individual parameters in Eq 1. 3) In complexes where a vast excess of ligand is used in the reaction, (k_on_[ligand] >> k_ex_),Eq 1 reduces to

$${\text{k}}_{\text{obs}}=\frac{{\text{K}}_{\text{D}}\times {\text{k}}_{\text{ex}}}{\left[\text{Pr}-\text{ligand}\right]}$$ (2)

In scenario 3, the complex reassociates before an H/D exchange event regardless of the estimated value of k_ex_. This analysis is equally relevant to protein-ligand interactions at orthosteric sites. Reporter amides at these sites would be sensitive to changes in H-bonding in the presence of saturating concentrations of ligand. It must be noted that these equations were originally described from deuterium off-exchange experiments [14].

Deuterium exchange is initiated by diluting an aqueous solution of protein in the presence of saturating concentrations of ligand in the equivalent buffer reconstituted in deuterium oxide (D_2_O). For ligands with known dissociation constants, it is necessary to use concentrations of ligand to ensure close to 100% binding to the target protein under deuterium exchange conditions. An additional factor that also is important is the high concentrations of ligand for k_on_*[ligand] >> k_ex_ (Eq 2). These conditions would facilitate preferential reassociation at the orthosteric binding site over deuterium exchange. Consequently, HDXMS experimental conditions for mapping high affinity inhibitor binding (radicicol (K_D_ = 19 nM) and 17-AAG (K_D_ = 33 nM)) (Table A in S1 Text), used at 20 μM (6: 1 ligand to target protein ratio). For the low affinity fragments, (Fragment **1** (K_D_ = 490 μM) and Fragment **2** (K_D_ = 570 μM), concentrations of fragments under deuterium exchange conditions were maintained at 5 mM (~ 1500:1 ligand to target protein ratio).

### Amide hydrogen/deuterium exchange mass spectrometry

Amide exchange reaction was initiated by adding 1 μL of 100 μM stock solution of Hsp90 protein in 27 μl of 99.9% D_2_O buffer (20 mM HEPES (4-(2-hydroxyethyl)-1-piperazineethanesulfonic acid), 300 mM NaCl, 10% (v/v) glycerol, 0.5 mM TCEP (tris(2-carboxyethyl)phosphine), pH 7.5) and 2 μL of ligand in dimethyl sulfoxide (DMSO) (for ligand binding) or DMSO (for ligand-free Apo) resulting in a final D_2_O concentration of 90% and Hsp90 concentration of 3.3 μM. Radicicol and 17-AAG were maintained at a final concentration of 20 μM while Fragments **1** and **2** were maintained at a final concentration of 5 mM. A list of all ligands tested along with their molecular weight and K_D_ values are given in Table A in S1 Text. HDXMS experimental set up, peptide identification and deuterium exchange data analysis were carried out as previously described [29] and is detailed in supplementary information.

## Results

### HDXMS-based heat map of Hsp90

**Fig 2:**
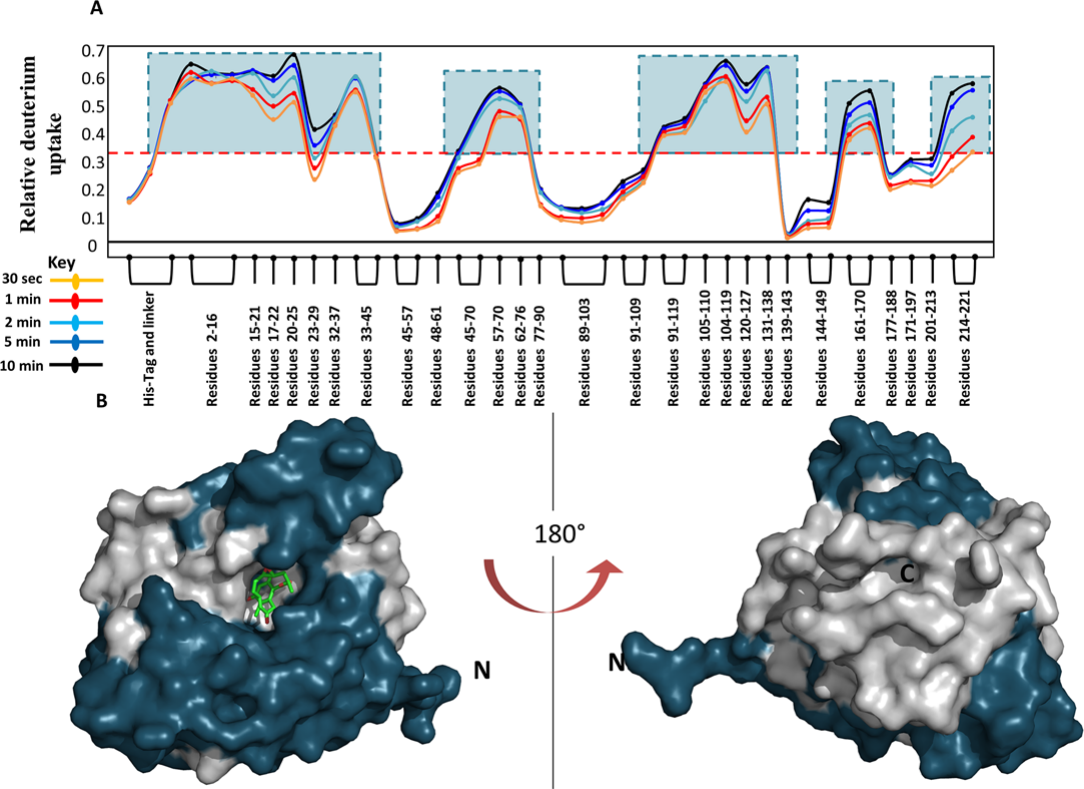
**(A)** A modified mirror plot of relative deuterium uptake values (y-axis) for every pepsin digest peptide analyzed, listed from the N to C terminus (x-axis) of Hsp90 for each time point of deuterium exchange. Reporter Regions were determined according to Eq 1 and boxed in teal. **(B, C)** Regions showing high dynamics were mapped onto the surface representation of Hsp90 (PDB Id: 4EGK) in blue bound to Radicicol (green sticks).

HDXMS experiments of ligand-free (*apo*) Hsp90 were carried out as described in materials and methods to generate a heat-map of dynamics at various loci in Hsp90. A total of 42 peptides were obtained corresponding to a sequence coverage of 95% of the primary sequence of Hsp90. Deuterium exchange values were quantified for each peptide as previously described [29] and each peptide was plotted against its relative deuterium uptake value in a relative exchange plot (Fig 2A). Here, the relative deuterium uptake (RDU) is the ratio of deuterons exchanged to exchangeable amides for each pepsin-digest fragment peptide listed from the N- to C-terminus for all deuterium exchange times (Fig 2A). RDU represents a readout of the relative dynamics at peptide resolution across the protein. The relative exchange plot of Hsp90 indicates multiple regions with RDU > 0.6 after a short deuteration time of 10 min. Here, we propose that RDU values greater than half the absolute maximum exchange observed after short deuterium labeling times can be used to identify peptide reporters for monitoring ligand interactions.

Reporter region if:RDU>0.5×maximum RDU for the protein for short labeling times (3)

For Hsp90, the maximum RDU value was calculated to be 0.67, thus regions with RDU values greater than 0.335 were characterized as reporters for monitoring ligand interactions (highlighted by blue boxes in Fig 2A). A map of these reporter peptides on the structure is shown in Fig 2B.

### Identifying orthosteric protein-ligand interactions in Hsp90

**Fig 3:**
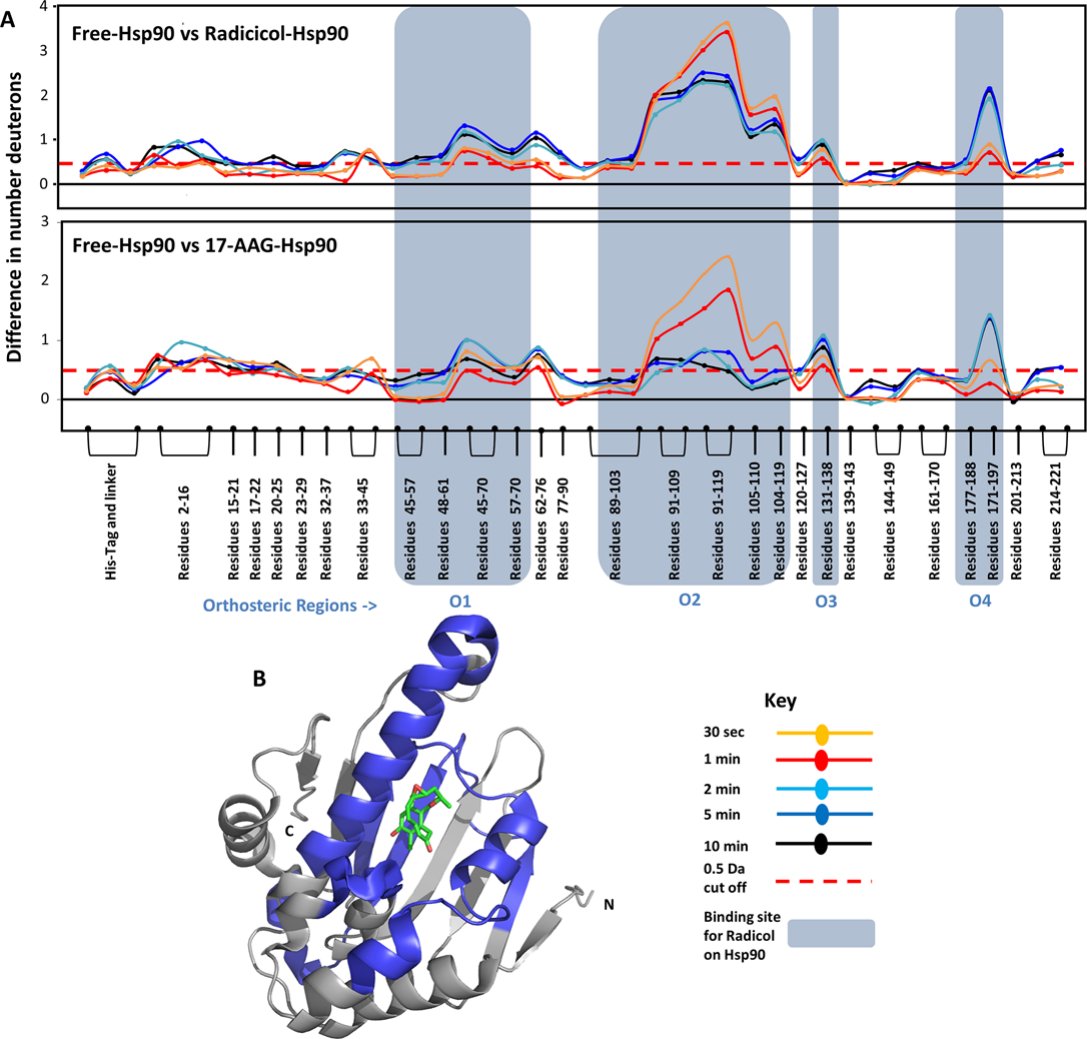
**(A)** The absolute difference in numbers of deuterons (inferred from difference in mass in Daltons (Da) (y-axis) between the free and ligand bound state is plotted for each pepsin digest fragment listed from the N to C terminus (x-axis) of Hsp90 for each Deuterium exchange time point (t = 0.5, 2, 5, 10 min) in a ‘difference plot’. Shifts in the positive scale represent decreases in deuterium exchange and shifts in the negative scale represent increases in deuterium exchange when compared to the apo-Hsp90. The top panel shows regions showing differences upon Radicicol binding and the bottom panel shows differences upon 17-AAG binding. Regions showing significant differences above a threshold of 0.5 Da (red dashed line) are compared with structural data to identify orthosteric regions. Peptides spanning these ligand binding sites which mediate orthosteric interactions with ligands are marked in blue boxes and are divided into four orthosteric regions marked O1 to O4.Time points of deuterium exchange are colored according to key. **(B)** Regions showing decreased exchange upon ligand binding (either Radicicol or 17-AAG) are mapped onto the structure of Hsp90 in blue. Radicicol bound at the ligand binding pocket is shown as sticks (PDB ID: 4EGK).

Effects of two high affinity ligands, Radicicol, a natural antibiotic, and 17-AAG, a Geldanamycin derivative, on Hsp90 were examined by HDXMS. These ligands bind Hsp90 with high affinities with binding constants (K_D_) in the nanomolar range (Table A in S1 Text). A difference plot of ligand-free Hsp90 and ligand-bound Hsp90 was used to determine differences in H-bonding and solvent accessibility in response to ligand binding (Fig 3A). Positive shifts indicate regions showing decreased deuterium exchange upon ligand binding and negative shifts indicate regions showing increased exchange are depicted using a negative axis. Both high affinity ligands tested showed decreased deuterium exchange across various regions in Hsp90. Binding of both Radicicol and 17-AAG caused decreased deuterium exchange at the same loci. There are seven distinct regions which show these changes. Upon close examination with structures of Hsp90 bound to radicicol (PDB ID:4EGK [30]) and 17-AAG (PDB ID: 1YET[31]), it is clear that both Radicicol and 17-AAG make multiple contacts with Hsp90. HDXMS analysis is sensitive to all these changes and peptides spanning these residues show significant changes upon ligand binding. In comparison with structural information of Hsp90, peptides spanning region O1 (residues 45 to 70) showed decreases in deuterium uptake due to contacts made by residue L48, N51, D54, A55 and K58. Peptides spanning region O2 (residues 89–119 making contacts at D93, I96, M98, D102, N106, L107 and K112), region O3 (residues 131–138 interacts with ligand at G135, V136, G137 and F138) and region O4 (residues 171–197 interact with ligand at T184 and V186) also showed decreases in deuterium exchange. HDXMS reporters (peptides) at these regions are highly sensitive to the orthosteric interactions made by Hsp90 to both Radicicol and 17-AAG [30]. It is clear that these spatially contiguous regions together constitute the site for orthosteric binding of ligands in Hsp90 (Fig 3B) and is identified with structures of ligand-bound Hsp90 [32, 33]. It is interesting that region O2 (89–119) shows larger magnitude differences in deuterium uptake at early time-points (0.5 min) while other orthosteric regions (O1,3,4) begins to show such changes only at or after 5min of exchange. The largest magnitude shifts in deuterium exchange are also observed in region O2, which contains the Asp93 residue known to hydrogen bond with Radicicol [30] and 17-AAG [31]. The other regions contain charged residues (Leu48, Asn51, Asp54, Ala55, Lys58, Ile91, Asp93, Ile96, Gly97, Met98, Asn106, Leu107, Lys112, Gly135, Phe138, Thr184, and Val186) that line the otherwise primarily hydrophobic binding pocket [31]. It is clear, by correlating HDXMS to structural data, that these regions form the orthosteric binding pocket for these two high affinity ligands. The region O2 which forms the direct H-binding contacts with the ligand showed the highest protection upon ligand binding. Our HDXMS results were able to identify all the important orthosteric contact points and highlights its powerful applicability for ligand screening.

### Low affinity fragments 1 and 2 bind at the same loci as the high affinity ligand

**Fig 4:**
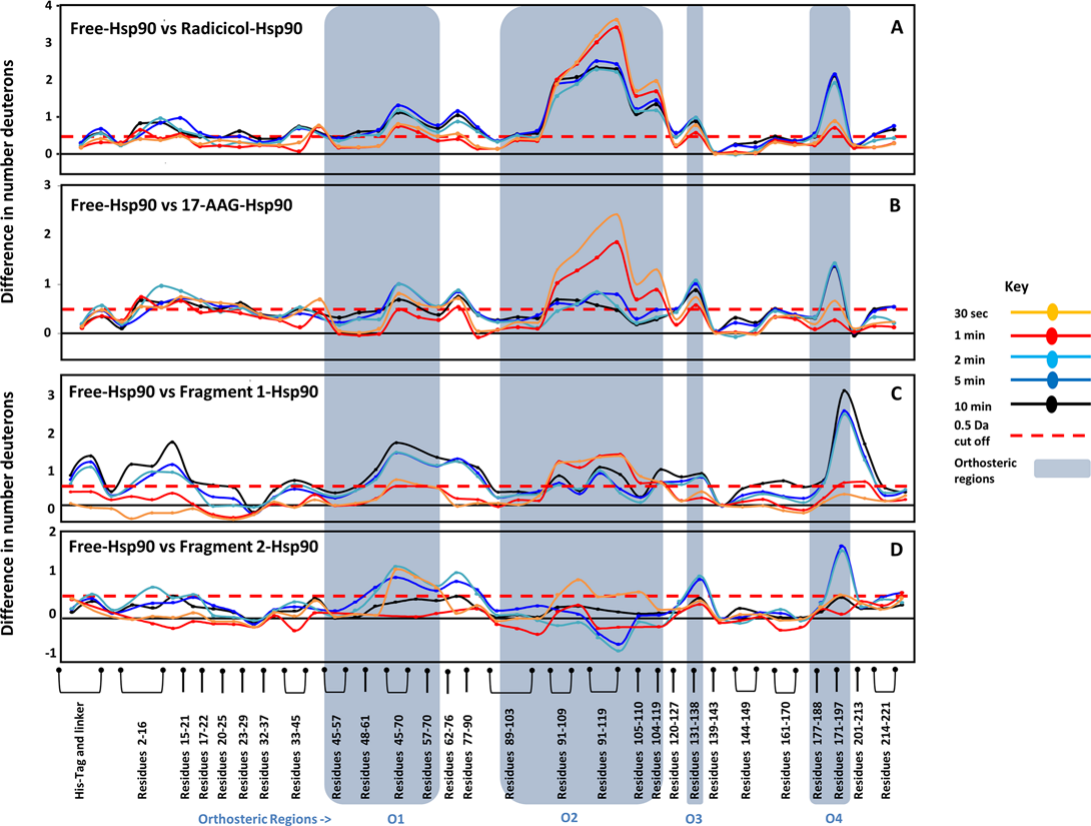
**(A)** Orthosteric regions identified by structural and HDXMS analysis overlap with changes in both ligands and fragments. The absolute difference in numbers of deuterons (y-axis) between the free and ligand bound state is plotted for each pepsin digest fragment listed from the N to C terminus (x-axis) of Hsp90 for each deuterium exchange time point (t = 0.5, 2, 5, 10 min) in a ‘difference plot’. Shifts in the positive scale represent decreases in deuterium exchange and shifts in the negative scale represent increases in deuterium exchange when compared to apo form of Hsp90. The panels A to D show regions showing differences upon binding Radicicol, 17-AAG, fragments **1** and **2**, respectively. Regions showing significant differences above a threshold of 0.5 Da (red dashed line) are correlated with structural data to define orthosteric sites. Peptides spanning these ligand binding sites which make orthosteric interactions with fragments are marked in blue boxes are divided into the same four orthosteric regions O1 to O4, observed in Radicicol and 17-AAG. Fragment **2** shows minimal protection in the region O2 as it is oriented away in this region, consistent with deuterium exchange data.

To test if low affinity fragment interactions share the same orthosteric binding site, two fragment molecules were used to test for binding with Hsp90 (grey box, Table A in S1 Text). These fragments are small phenolic compound derivatives < 300 Da with low affinities (K_D_ ~0.5mM) and make less than 3 hydrogen bonds [21, 26]. HDXMS experiments of Hsp90 interactions with these two fragment molecules, revealed distinct effects on deuterium exchange across Hsp90. Both fragments cause changes due to binding at the four orthrosteric regions observed in Radicicol and 17-AAG (Fig 4). Fragment **1** mediated more stable interactions (based on the extent of deuterium exchange protection) at the orthosteric regions and showed decreased deuterium exchange similar to the high affinity ligands, while fragment **2** only showed decreases at 0.5 min of deuteration in the region O2 (Fig 4C and 4D). This is consistent with insights from crystallographic structures of Hsp90 in complex with fragments **1** and **2**, where it is evident that fragment **1** makes contacts with the residues 109–119 in the region O2, while fragment **2** is oriented away from this region of the binding pocket [34].

All the orthosteric regions showed decreased deuterium exchange in the presence of both high affinity ligands and fragments **1** and **2**, however the magnitude difference with fragments was lower especially at region O2. Radicicol and 17-AAG binding resulted in a large decrease in deuterium exchange of up to 3 Da (Fig 4A and 4B), while fragments **1** and **2** showed a decrease in deuterium exchange of ~1 Da (Fig 4C and 4D). Other regions O1, O3 and O4 in the orthosteric pocket showed decreased exchange at later time points upon fragment binding, to the same extent seen with high affinity ligands.

High affinity ligands are large molecules with multiple active moieties that are stabilized by interactions with multiple residues in the binding pocket of the protein. In contrast, fragments being smaller molecules with fewer heavy atoms and can be expected to mediate fewer interactions in the ligand binding pocket. It is therefore interesting that the fragments tested showed effects at all loci that the high affinity ligands perturbed.

### Non-orthosteric distal changes in Hsp90 reveal allosteric responses to ligand binding

**Fig 5:**
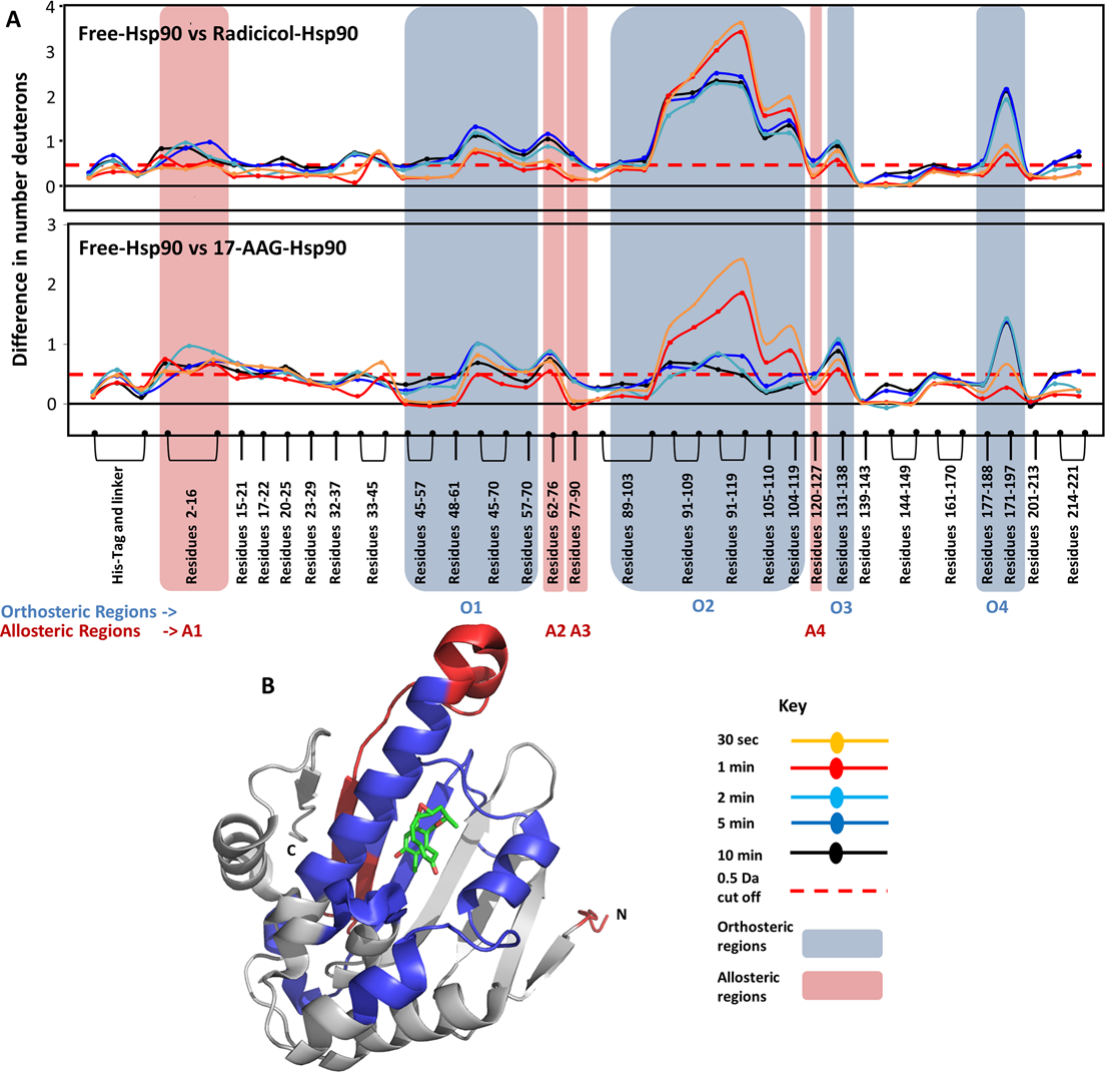
Distinguishing orthosteric and allosteric effects in Hsp90. **(A)** The absolute difference in numbers of deuterons (y-axis) between the free and ligand bound state is plotted for each pepsin digest fragment listed from the N to C terminus (x-axis) of Hsp90 for each deuterium exchange time point (t = 0.5, 2, 5, 10 min) in a ‘difference plot’. Shifts in the positive scale represent decreases in deuterium exchange and shifts in the negative scale represent increases in deuterium exchange when compared to apo-Hsp90. Regions showing significant differences above a threshold of 0.5 Da (red dashed line) are compared with orthosteric sites (blue boxes) to predict allosteric regions. Peptides highlighted in red show regions showing differences in distal allosteric regions, not involved in orthosteric binding. Peptides spanning these regions are marked in red boxes and divided into four allosteric regions A1 to A4. Radicicol and 17-AAG shows differences in A1 and A2, while only radicicol showed changes in A3 and A4. Time points are colored according to key. **(B)** Predicted allosteric regions are mapped on to the structure of Hsp90 (red), together with the orthosteric regions, in blue. Radicicol bound at the ligand binding pocket is shown as sticks (PDB ID: 4EGK).

It is clear from an overlay of HDXMS on the high resolution crystallographic structures of Hsp90-ligand and fragment complexes, that regions O1 to O4 form the orthosteric binding pocket for high affinity ligands and fragment molecules. Significantly, there are additional regions which show decreases in deuterium uptake in ligand-bound Hsp90. Regions A1, A2 and A3 are distal to the binding pocket, yet show significant differences decreases in deuterium uptake upon addition of ligand (Fig 5A). These distal allosteric changes due to Radicicol and 17-AAG are regions A1 (residues 2–16) and A2 (residues 62–90). Radicicol shows additional allosteric effects at regions A3 (residues 77–90) and A4 (residues 120–127). Since there are no direct contacts mediated by the ligand at these regions, any changes observed would be a result of secondary stabilization or long-range conformational effects, and are strongly suggestive of allostery.

**Fig 6:**
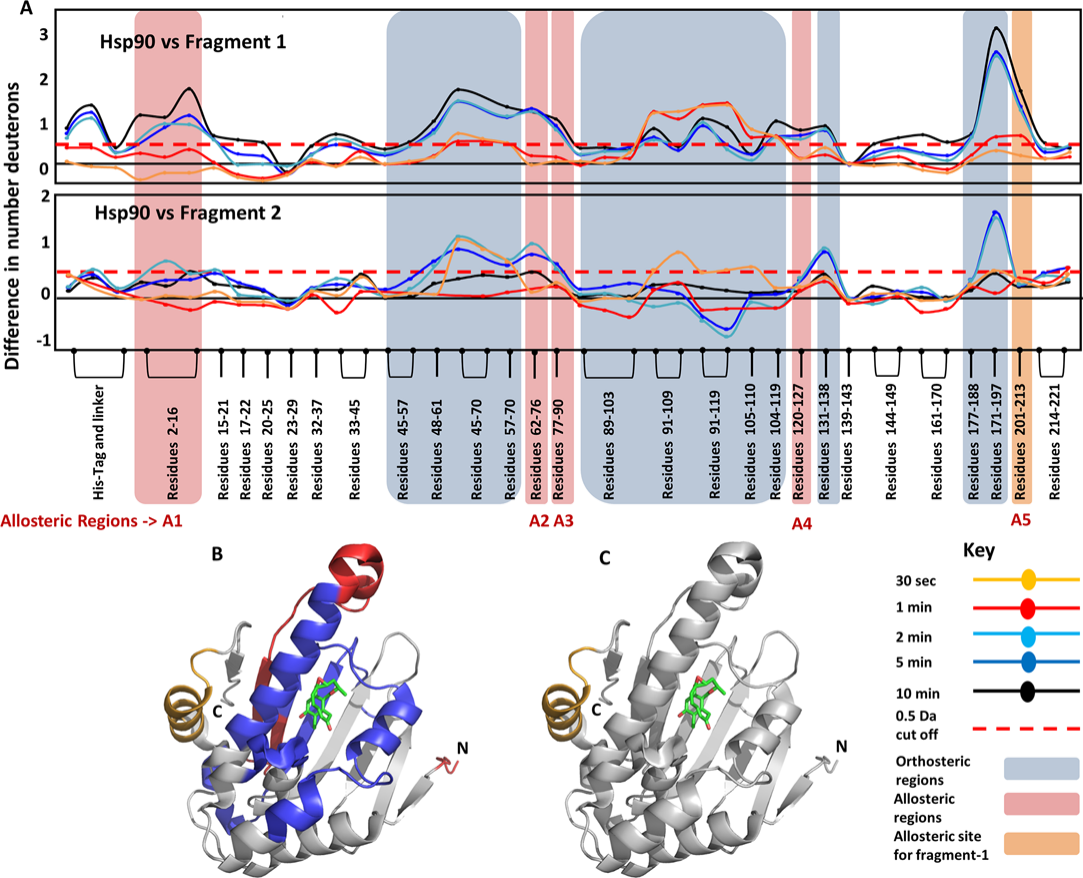
Fragments 1 and 2 differ in the nature of the allosteric effect in Hsp90. **(A)** The absolute difference in numbers of deuterons (inferred from difference in mass in Daltons (Da) (y-axis) between the free and ligand bound state is plotted for each pepsin digest fragment listed from the N to C terminus (x-axis) of Hsp90 for each deuterium exchange time point (t = 0.5, 2, 5, 10 min) in a ‘difference plot’. Shifts in the positive scale represent decreases in deuterium exchange and shifts in the negative scale represent increases in deuterium exchange when compared to the apo-Hsp90. Regions showing significant differences above a threshold of 0.5 Da (red dashed line) are compared with orthosteric sites (blue boxes) to establish allosteric regions (red boxed). Fragment **2** does not show any changes in region A4, similar to 17-AAG, while fragment **1** shows differences, similar to Radicicol. In addition, fragment **1** shows an allosteric response at the regions A5 (residues 201–213 shown in orange box), which is not observed in the other three ligands. Time points are colored according to key. **(B,C)** The identified orthosteric (blue) and allosteric regions (red) for fragments are mapped on to the structure of Hsp90 in blue. **(C)** The allosteric site A5 in Hsp90, which is observed only fragment **2** is highlighted in orange. Radicicol bound at the ligand binding pocket is shown as sticks (PDB ID: 4EGK).

Fragments also showed shifts in deuterium exchange in Hsp90 at the same regions (Fig 6). Although fragments **1** and **2** caused similar effects in orthosteric sites, there were significant differences in the allosteric responses that they elicit. Fragment **1** showed distal effects at regions A1, A2, A3 and A4, similar to Radicicol. In addition, Fragment **1** also showed an allosteric change at region A5. It was unexpected that the fragment **1** molecule would result in allosteric effects at a region unobserved with both high affinity ligands. This region is highlighted in orange in Fig 6A (top panel). This peptide is part of a helix region that is distal to the binding site and spans residues 201 to 213 (Fig 6C).

## Discussion

### HDXMS as a sensitive tool to probe interactions of low affinity ligands to proteins

While HDXMS has proven to be a powerful technique to monitor protein-ligand interactions; its role in mapping low affinity protein-fragment interactions is less known. Here, we have mapped and compared interactions of ATP binding domain of Hsp90 with two high affinity ligands; Radicicol and 17-AAG; and two low affinity phenolic fragment compounds: **1** and **2** by HDXMS. The results of HDXMS are entirely consistent with the high resolution structures of the protein-ligand complexes. This validates a potential application of HDXMS in screening high and low affinity compounds for drug discovery, Importantly, our results also show that the two fragment compounds exhibit distinct effects on deuterium exchange of Hsp90 measured by HDXMS, with fragments **1** and **2** showing differences in deuterium exchange protection in Hsp90 (Figs 4 and 6). The ability to differentiate between the effects of these two fragments on Hsp90 was striking as both **1** and **2** belong to the same phenolic class of fragment molecules and are similar in molecular size and affinities. These results highlight how HDXMS is a highly sensitive and robust method to distinguish subtle differences in interactions mediated by closely related compounds.

### Mapping orthosteric binding sites on Hsp90

The readout from HDXMS experiments encompasses changes upon binding at both the orthosteric site and long range conformational changes accompanying binding. Distinguishing these effects apart is a challenge made easier with the availability of high resolution structures of protein-ligand complexes. These structures provide a detailed architecture of the orthosteric binding site and the atomic contacts mediated by the ligand. However, structures obtained by X-ray crystallography, seldom provide any insights into long range conformational changes. This makes HDXMS a perfect complementary tool with X-ray crystallography to identify and map orthosteric binding sites and provide important additional insights into accompanying long range allosteric changes. In Hsp90, pepsin fragment peptides spanning regions O2 (residues 90–119) show the greatest magnitude decreases in exchange in the presence of radicicol and 17-AAG at all time points of exchange. This region overlaps with the structure of radicicol and 17-AAG bound to Hsp90 (Fig 3). Asp93 in this region forms a direct hydrogen bond with the ligands and is the basis for protection seen in HDXMS. The other orthosteric regions are made up of direct and polar contacts made by the ligands with residues that line the binding pocket. The magnitude differences in deuterium exchange are greatest at early time points of exchange and these differentially drop with increasing deuteration times, as a function of their individual dissociation rates (k_off_). A larger protection in deuterium exchange observed at region O2 in the radicicol-Hsp90 complex is observable over longer deuterium exchange times relative to the 17-AAG-Hsp90 complex. One study reports the kinetics of AAG-Hsp90 interactions with a dissociation rate of 0.3 min^-1^ [35]. At these dissociation rates, highest protection would be seen at earlier time points (0.5 min) with a reduction at longer time points as observed (Fig 3A bottom panel). We can additionally glean important insights and differences between the two high affinity ligands from the kinetic data. Based on the retention of protection factors for longer deuteration time points at this locus in the presence of radicicol, we predict a slower k_off_ for radicicol compared to 17-AAG, if the k_on_ are assumed to be equivalent.

Analysis of the effects of fragments on the deuterium exchange across Hsp90 showed interestingly both fragments showed protection at the same loci (regions O1 to O4) and these align with the binding pocket identified in the crystal structure of the ligand-Hsp90 complex. This structural locus thus represents the orthosteric binding site for both high affinity ligands and fragments. The magnitude protection in the presence of Fragments **1** and **2** at this site was lower and this is consistent with the fewer expected H-bonds mediated by smaller fragment molecules (Fig 4).

### Changes in regions outside orthosteric site: Long-range conformational changes with allosteric implications

One of the strengths and challenges with HDXMS are its exquisite sensitivity which generates a comprehensive set of changes, attributable to a range of protein motions such as inherent fluctuations, local unfolding, domain motions and conformational rearrangements, upon ligand binding. It is relatively straightforward to identify allostery and localize allosteric sites when increases in deuterium exchange in regions of the protein, in response to binding at a noncontiguous site, are observed [36]. However it is a major challenge for interpretation when there are multiple noncontiguous sites on a target protein showing decreases in deuterium exchange in response to ligand binding as it can be unclear which of the changes are attributable to binding site (orthosteric) interactions and which reflect long range conformational changes at distal sites. In this study we report how HDXMS is uniquely poised to allow a separation of effects at the binding site from changes due to long range conformational changes by examining regions outside the orthosteric binding sites and validated by X-ray crystallography.

Although the orthosteric and allosteric changes are spatially distinguishable, there is no specific time-dependent pattern observed in both the changes. Region O2 shows early time-point changes while O1, O3 and O4 only show changes at longer deuteration time-points (Fig 5). It must be noted that the protection observed at a particular locus is dependent upon the intrinsic deuterium exchange rate (k_ex_). Unlike unfolded oligopeptides/ short polypeptides, it is clear that different amide-hydrogens have different rates of intrinsic H/D exchange (k_ex_). Even within a single peptide, there could be a combination of fast and slow exchanging amides. These intrinsic rates of exchange largely determine the deuterium uptake with respect to time. It is also seen that protection due to H-bonding interactions generally show up in early time-scales due to the protection of the amide by the ligand. So, it is also possible that the region O2 shows large decreases in early time-points due to specific H-bonding interactions between ligand and Asp93.

In contrast, at the allosteric site there is no such effect of H-bonding or solvent exclusion by ligand and the rate of observed deuterium exchange is solely dependent on the rate of conformational change and rate of intrinsic exchange (k_ex_) at the allosteric site. Again, the changes in deuteration apparent at later deuteration time-points is largely a consequence of slower rates of intrinsic exchange (k_ex_) at these loci compared to the rates of conformational change. All of the allosteric regions predicted in Hsp90 show decreases at longer deuteration times. It is possible that allosteric regions predominantly contain slower exchanging amides in general or this could be unique to Hsp90. In order to validate either or both these hypotheses, larger sets of protein-ligand systems would need to be examined.

Applications of HDXMS to distinguish direct ligand binding effects from other conformational effects in interactions between the bacteriophage HK97 and its processing protease have been reported when HDXMS is combined with high resolution structural biology tools [37, 38]. Here we have shown that it is possible to apply HDXMS to distinguish direct ligand binding effects from other effects even when monitoring weak affinity protein-ligand interactions.

### Distinguishing fragment interactions: Implications for fragment-based drug design

Our results (Fig 4) show that every fragment is unique in the changes it induces in the protein both as a consequence of binding and associated long range conformational changes. Differences in magnitude of deuterium exchange protection can be used to determine fragment efficacy. In the case of Hsp90, fragment **2** mediates weaker interactions at the binding pocket compared to fragment **1.** Based on this observation we predict faster dissociation rates for fragment **2** relative to fragment **1,** assuming comparable association rates. Fragments can be ranked based on both the number of regions showing differences in deuterium exchange and the magnitude shifts and offers a framework for iterative fragment expansion and coupling in the FBLD pipeline. By mapping binding sites of low affinity ligands to proteins in solution, HDXMS offers a powerful complement to X-ray crystallography.

## Supporting Information

S1 Text Supporting information describes the properties of the high and low affinity ligands used in this study in Table A. Purification of the N-terminal ATPase domain of Hsp90 are described together with details of the amide hydrogen/deuterium exchange mass spectrometry experiments.
(DOCX)
