# Supplementary material for: Predicting Allosteric Effects from Orthosteric Binding in Hsp90-Ligand Interactions: Implications for Fragment-Based Drug Design
Source: PLoS Comput Biol. 2016 Jun 2;12(6):e1004840. doi: 10.1371/journal.pcbi.1004840 (PMC4890749; doi:10.1371/journal.pcbi.1004840)
Supplement: S1 Text — Purification of the N-terminal ATPase domain of Hsp90 are described together with details of the amide hydrogen/deuterium exchange mass spectrometry experiments. (DOCX) [file pcbi.1004840.s001.docx]

**S1 Text**

Predicting allosteric effects from orthosteric binding in Hsp90-ligand interactions: Implications for Fragment-based drug design

*Arun Chandramohan^1^***, Srinath Krishnamurthy^1^***, Andreas Larsson^2^, Paer Nordlund^2^, Anna Jansson^2^ and Ganesh S. Anand^1^*

^1^ Department of Biological Sciences, National University of Singapore, 14 Science Drive 4, Singapore 117543

*^2^* School of Biological Sciences, Nanyang Technological University, 61 Nanyang Drive, Singapore 639798

**Abstract**

Supporting information describes the properties of the high and low affinity ligands used in this study in Table A. Purification of the N-terminal ATPase domain of Hsp90 are described together with details of the amide hydrogen/deuterium exchange mass spectrometry experiments.


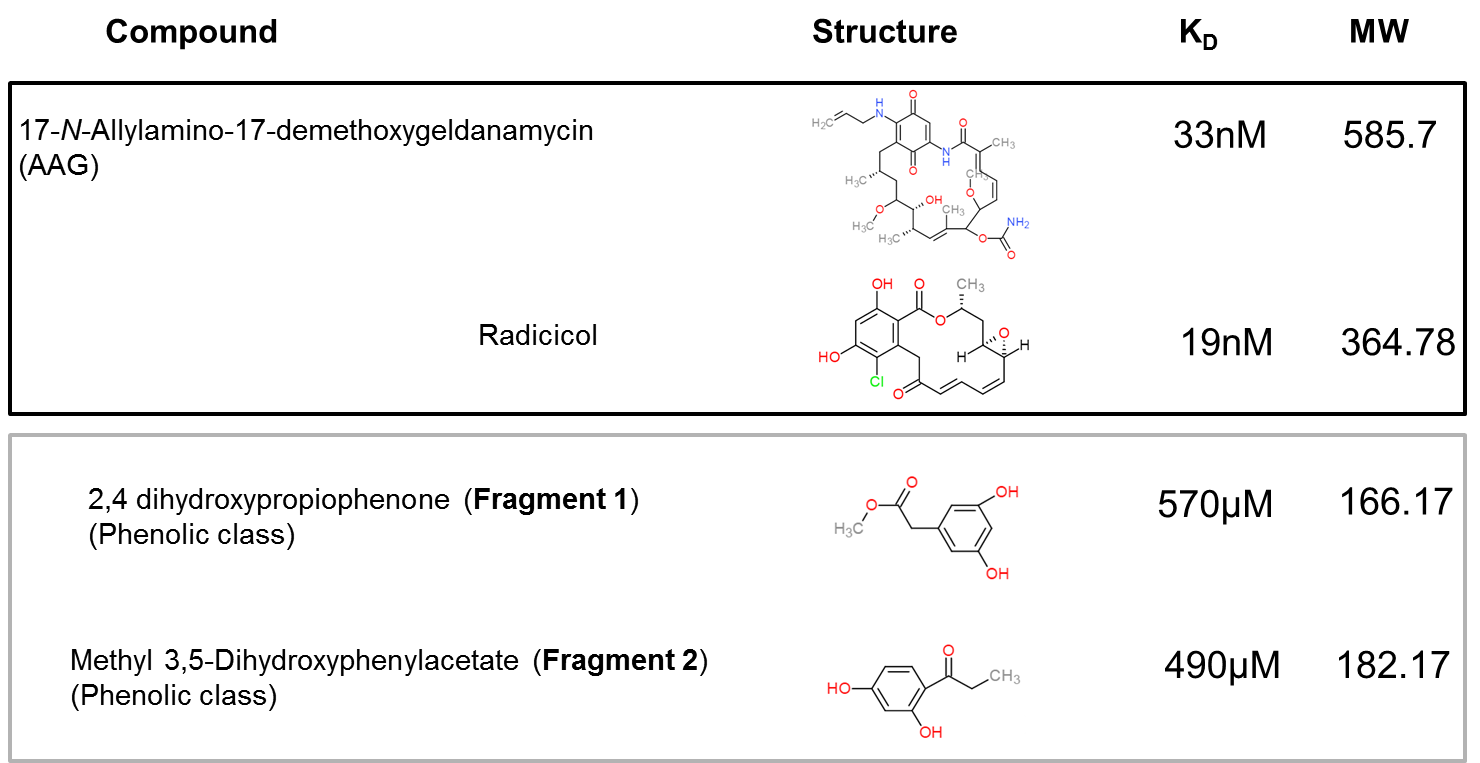


**Table A:** Ligands tested for binding to Hsp90 by HDXMS along with their molecular weights and binding affinities. High affinity ligands are boxed in black and low affinity ligands boxed in grey [27,28].

**Materials**

Unless otherwise mentioned all ligands/compounds were purchased from Sigma Aldrich (St. Louis, MO).

**Protein Expression and Purification**

The gene coding for M1-E236, the ATP binding domain of human Hsp90 isoform 1, were subcloned into the pNIC28-Bsa4 vector (GenBank^TM^ accession number EF198106), yielding an expression construct with an N-terminal hexahistidine tag and a TEV protease recognition site. The positive recombinant clone was retransformed and expressed in T1 phage-resistant BL21(DE3) *E. coli* strain (Merck). For expression, the cells were grown at 37°C in a LEX system using 0.75 L of Terrific Broth medium supplemented with 8 g/L glycerol, 50 μg/mL of kanamycin, and 34 μg/mL of chloramphenicol. When OD_600_ reached 0.6-0.8, the temperature was reduced to 18 °C. After 30–60 min, the expression of the target protein was induced by addition of 0.5 mM isopropyl β-D-thiogalactopyranoside (IPTG) and incubated for 17–20 h. The cells were harvested by centrifugation and resuspended in lysis buffer (100 mM HEPES, 500 mM NaCl, 10 mM imidazole, 10% (v/v) glycerol, 0.5 mM TCEP, pH 8.0) supplemented with Protease Inhibitor Mixture Set III, EDTA free (Merck) and 2000 units of benzonase (Merck), and stored at −80 °C. Cells were disrupted by sonication on ice using Vibra-Cell processor (Sonics & Materials Inc., Newtown, CT). The lysate was clarified by centrifugation at 47,000 × *g* for 25 min at 4 °C, and the supernatant was filtered through a 1.2-μm syringe filter. Filtered lysates were loaded onto 1 mL of nickel-nitrilotriacetic acid Superflow resin (Qiagen Inc., Valencia, CA) in IMAC wash buffer 1 (20 mM HEPES, 500 mM NaCl, 10 mM imidazole, 10% (v/v) glycerol, 0.5 mM TCEP, pH 7.5) and washed with IMAC wash buffer 2 (20 mM HEPES, 500 mM NaCl, 25 mM imidazole, 10% (v/v) glycerol, 0.5 mM TCEP, pH 7.5). Bound proteins were eluted with 500 mM imidazole and loaded onto a HiLoad 16/60 Superdex-200 column (GE Healthcare, Waukesha, WI) pre-equilibrated with equilibration buffer (20 mM HEPES, 300 mM NaCl, 10% (v/v) glycerol, 0.5 mM TCEP, pH 7.5). Fractions containing the protein of interest were pooled. TCEP was added to a final concentration of 2 mM, and the sample was concentrated using Vivaspin 20 filter concentrators (15-kDa MW cutoff) (GE Healthcare) at 15 °C. The final protein concentration and yield were 16.5 mg/mL, 6 mg.

**Experimental optimization and design**

The sensitivity of mass spectrometers currently available can easily detect picomole-femtomole amounts of protein sample. The sensitivity limit of the mass spectrometer can be used to guide the amount of protein sample used for the deuterium exchange reaction.

For ligands where dissociation constants are unknown, highest concentration solutions of ligand achievable in DMSO without precipitation may be used.

Due to the variable aqueous solubility of ligands from compound libraries, dimethyl sulfoxide (DMSO) is a preferred solvent. An alternate approach is for the ligand solubilized in DMSO to be added to the deuterated buffer and the deuterium exchange reaction initiated by addition of protein to offset potentially adverse interactions of protein with higher concentrations DMSO prior to dilution in D_2_O. The protein sample of interest should be tested through functional assays to ensure that it can tolerate the final concentration of DMSO present in the deuterium exchange reaction mixture.

**Amide Hydrogen/Deuterium Exchange Mass Spectrometry**

Briefly, deuterium exchange reaction was carried out for the following time points (t= 0.5, 1, 2, 5 and 10 min). This time series represents a short labeling time series for deuterium exchange [39]. The reaction was quenched in 2% TFA solution to obtain a pH_Read_ of ~2.5 and chilled on ice for 2 min prior to injection in a nano-UPLC HDX sample manager (Waters, Milford, MA) [40]. Samples were subjected to proteolytic digestion by an online immobilized pepsin column and separated on C18 analytical reverse phase column. Peptides were then sprayed onto a Waters Synapt HDMS ESI mass spectrometer (Waters, Milford, MA) acquiring in MS^E^ mode. Continuous instrument calibration was carried out using Glu-fibrinogen peptide. Reported values are the average of 2-4 independent experiments. We have previously reported an average back exchange of ~ 30% in our experimental setup [41]. In this study, only uncorrected deuterium exchange values are reported.

Pepsin digest fragments were sequenced and identified using ProteinLynx Global Server version 2.0 (Waters, Milford, MA) and deuterium exchange data was analyzed using DynamX software version 2.0 (Waters, Milford, MA).

Supporting references

(39) Hoofnagle, A. N.; Resing, K. A.; Ahn, N. G. *Annu Rev Biophys Biomol Struct* **2003**, *32*, 1-25.

(40) Wales, T. E.; Fadgen, K. E.; Gerhardt, G. C.; Engen, J. R. *Anal Chem* **2008**, *80*, 6815-6820.

(41) Moorthy, B. S.; Gao, Y.; Anand, G. S. *Mol Cell Proteomics* **2011**, *10*, M110 002295.
